# Emergence of *Streptococcus pneumoniae* Serotype 12F after Sequential Introduction of 7- and 13-Valent Vaccines, Israel

**DOI:** 10.3201/eid2403.170769

**Published:** 2018-03

**Authors:** Assaf Rokney, Shalom Ben-Shimol, Zinaida Korenman, Nurith Porat, Zeev Gorodnitzky, Noga Givon-Lavi, Merav Ron, Vered Agmon, Ron Dagan, Lea Valinsky

**Affiliations:** Ministry of Health, Jerusalem, Israel (A. Rokney, Z. Korenman, Z. Gorodnitzky, M. Ron, V. Agmon, L. Valinsky);; Soroka University Medical Center, Beer Sheva, Israel (S. Ben-Shimol, N. Porat, N. Givon-Lavi);; Ben-Gurion University of the Negev, Beer Sheva (S. Ben-Shimol, N. Porat, N. Givon-Lavi, R. Dagan)

**Keywords:** Streptocococcus pneumoniae infections, 12F polysaccharides Streptocococcus pneumoniae, 7-valent pneumococcal vaccine, 13-valent pneumococcal vaccine, emerging infectious diseases, Israel, bacteria, vaccines, immunization

## Abstract

Israel implemented use of 7- and 13-valent pneumococcal vaccine in 2009 and 2010, respectively. We describe results of prospective, population-based, nationwide active surveillance of *Streptococcus pneumoniae* serotype 12F (Sp12F) invasive pneumococcal disease (IPD) dynamics in the 7 years after vaccine introduction. Of 4,573 IPD episodes during July 2009–June 2016, a total of 434 (9.5%) were caused by Sp12F. Sp12F IPD rates (cases/100,000 population) increased in children <5 years of age, from 1.44 in 2009–2010 to >3.9 since 2011–2012, followed by an increase in all ages. During 2011–2016, Sp12F was the most prevalent IPD serotype. Sp12F isolates were mostly penicillin nonsusceptible (MIC >0.06 µg/mL; MIC_50_ = 0.12) and predominantly of sequence type 3774), a clone exclusively found in Israel (constituting ≈90% of isolates in 2000–2009). The sharp increase, long duration, and predominance of Sp12F IPD after vaccine implementation reflect a single clone expansion and may represent more than a transient outbreak.

*Streptococcus pneumoniae* is a leading cause of illness and death worldwide; the highest incidence occurrs in children <2 years of age and in the elderly (*1*–*5*). For this reason, pneumococcal diseases are a target for global immunization programs in children and adults (*1*,*6*,*7*). Most reported cases of invasive pneumococcal disease (IPD) are sporadic, and outbreaks occur infrequently (*5*). Reported outbreaks have been of limited epidemiologic significance and associated with specific serotypes, including *Streptococcus pneumoniae* serotype 12F (Sp12F) (*5*,*8*,*9*).

Since the introduction of pneumococcal conjugated vaccine (PCV), a substantial increase in nasopharyngeal carriage of nonvaccine serotypes (NVT) has been observed in surveillance studies (*10*–*14*) and randomized trials assessing the effect of PCV on NVT carriage, comparing vaccinated children and unvaccinated controls (*10*). This increase in NVT carriage, commonly referred to as the replacement phenomenon, was accompanied by an increase in rates of IPD caused by NVT, diminishing the overall impact of PCVs on IPD rates (*10*,*11*).

In Israel, the 7-valent PCV (PCV7) was introduced to the national immunization plan (NIP) in July 2009, with a catch-up plan for children <2 years of age, and was gradually replaced with the 13-valent PCV (PCV13) in November 2010, without further catch-up. Uptake rates were high (>90%) (*14*). PCV7/PCV13 sequential introduction was followed by a substantial decline in overall IPD incidence among all age groups, driven mainly by the near-elimination of vaccine serotype disease, but accompanied by a substantial increase in IPD caused by non-PCV13 serotypes (*15*,*16*).

Sp12F is a nonvaccine serotype that is uncommonly carried in healthy persons (*12*,*13*,*17*). Sp12F was relatively rare in Israel among IPD cases in the pre-PCV era but has increased noticeably following PCV introduction (*15*). This serotype has been associated with outbreaks of community-acquired pneumonia and IPD in the United States (*17*–*20*) and Canada (*8*,*21*). Globally, most reported Sp12F isolates were of sequence type (ST) lineages ST989 and ST218 (*17*,*18*,*22*,*23*). An outbreak clone in Canada has been reported to have acquired resistance to macrolides and fluoroquinolones, replacing susceptible clones (*21*), but in general Sp12F is penicillin susceptible.

We assessed the dynamics of Sp12F IPD rates after sequential introduction of PCV7/PCV13 in Israel. Additionally, we investigated the molecular and antimicrobial drug susceptibility characteristics of these serotype isolates before and after vaccine introduction.

## Materials and Methods

### Study Design

Our data derive from ongoing nationwide, prospective, population-based, active surveillance on IPD in children and adults in Israel (*15*,*16*). This report concentrates on data collected during the 7 years that followed PCV introduction in Israel (July 2009–June 2016). The study was conducted in all 27 medical health centers routinely obtaining cerebrospinal fluid (CSF) and blood cultures from children and adults: 26 hospitals and 1 major outpatient health maintenance organization. Less than 1% of blood cultures and no CSF cultures were obtained outside these centers. This setting enabled us to cover all culture-confirmed IPD cases among the population of Israel (*15*).

IPD isolates are sent regularly to the national reference center at the Central Laboratories of the Ministry of Health in Jerusalem for confirmation and serotyping (*15*,*16*). Since 2009, active surveillance on IPD in all ages has been conducted in all 27 laboratories performing blood cultures in Israel. The capture–recapture method ensured the reporting of >95% of cases. Using these data, we completed the isolates missing from the passive surveillance system, so that all *S. pneumoniae* isolates from blood, CSF, or both collected during the relevant period in Israel were included in this study. Eventually, *S. pneumoniae* strains from 4,573 IPD cases, isolated from blood or CSF, were included in the study. The population breakdown during the study period is given in Table 1. During this period, the Jewish population was ≈79% of the total population (*24*).

**Table 1:** Age distribution of case-patients with of Sp12F infection, Israel, July 2009–June 2016*

Year	Age <5 y			Age 5–17 y			Age 17–64 y			Age >65 y	
	No. cases	Total population		No. cases	Total population		No. cases	Total population		No. cases	Total population
Jul 2009–Jun 2010	11	763,700		1	1,706,200		3	4,341,250		5	743,250
Jul 2010–Jun 2011	17	784,000		2	1,737,250		13	4,405,100		9	768,100
Jul 2011–Jun 2012	38	804,750		1	1,768,700		16	4,465,500		14	799,000
Jul 2012–Jun 2013	36	824,300		2	1,802,100		17	4,526,750		13	831,750
Jul 2013–Jun 2014	48	841,400		2	1,841,100		15	4,589,000		12	866,050
Jul 2014–Jun 2015	34	864,000		4	1,904,700		32	4,692,000		18	919,600
Jul 2015–Jun 2016	42	885,600		2	1,947,100		13	4,765,500		14	959,650
Total no. cases	226			14			109			85	

*Sp12F, *Streptococcus pneumoniae* serotype 12F.

### Case Definition

We defined an IPD episode by isolation of *S. pneumoniae* from blood or CSF. We excluded positive cultures from sterile sites other than blood or CSF (i.e., joint/pleural fluid or peritoneal fluid) (*15*,*16*), as well as diagnoses based solely on nonculture methods (PCR, antigen testing, Gram stain, or clinical diagnosis only).

### Vaccine Uptake

The method of evaluating vaccine uptake initiated in July 2009 has been described elsewhere (*14*,*15*). In June 2009, the proportion of children 12–23 months of age who received >2 of any PCV doses was 20%; this proportion increased to 71% in June 2010 and has increased to ≈95% since 2011.

### Bacteriology

We inoculated *S. pneumoniae* cultures onto tryptic soy agar plates supplemented with 5% defibrinated sheep blood (Hy-labs, Rehovot, Israel) and incubated them at 37°C for 24 hours in a 5% enriched CO_2_ atmosphere. Identification of *S. pneumoniae* was done as previously described (*15*).

### Serotypes

All strains were serotyped by the Quellung test; since 2013, the serotype has been determined by a validated combination of the capsular sequence typing molecular typing protocol and serotyping using the antisera of Statens Serum Institute (Copenhagen, Denmark). Serotype 6A was differentiated from serotype 6C by PCR (*10*).

### Antimicrobial Drug Susceptibility Testing

We determined susceptibility to penicillin for Sp12F strains by the oxacillin disk diffusion screening method of Bauer and Kirby and Etest (BioMérieux, Marcy l’Étoile, France) according to Clinical and Laboratory Standards Institute (CLSI) guidelines (*25*). Because Sp12F often causes meningitis, we set the susceptibility cutoff values according to the CLSI cutoff for meningitis. Thus, we defined isolates with penicillin MICs of <0.06 μg/mL as susceptible to penicillin and considered those with MICs >0.06 μg/mL to be nonsusceptible.

### Pulsed-Field Gel Electrophoresis 

We determined the genetic relatedness for all IPD strains isolated since 2009 and all Sp12F strains isolated during 2000–2008 available in our strains bank. We prepared and analyzed chromosomal DNA fragments generated by *Sma*I digestion as described previously (*11*,*26*,*27*), with modifications.

We performed genotype analysis and clustering using the Bionumerics version 7.6 software package (Applied-Maths, Sint-Martens-Latem, Belgium) with dice coefficients, a 1% position tolerance, and optimization values. We performed cluster analysis by the unweighted pair-group mean analysis. All isolates in the same cluster, defined as clonal, were assigned a letter, A to T, for analysis purposes.

### Multilocus Sequence Typing 

We characterized selected isolates representing pulsed-field gel electrolysis (PFGE) cluster and isolate years by multilocus sequence typing (MLST). The 7 housekeeping loci (*aro*E, *ddl, gdh, gki, rec*P, *spi,* and* xpt*) were amplified according to the *S. pneumoniae* PubMLST (*28*). We crudely extracted bacterial cells by boiling and performed PCR and sequencing by using a revised protocol provided by Bruno Pichon (Antimicrobial Resistance and Healthcare Associated Infection Unit, Public Health England, pers. comm., 2016 Sept 16).

We designed new primers for allele amplification and sequencing. These primers do not contain degenerative bases; an M13 tag on each 5′ primer sequence was added to ease sequencing setup (Table 2). Sequencing of the forward and reverse amplicons was performed at the Center for Genomic Technologies, Institute of Life Sciences, The Hebrew University of Jerusalem (Jerusalem, Israel), using BigDye Terminator v1.1 chemistry (Applied Biosystems, Foster City, CA, USA).

**Table 2:** Multilocus sequence typing PCR amplification and sequencing primers for study of *Streptococcus pneumoniae* serotypes, Israel, July 2009–June 2016

Primers	Sequence, 5′ → 3′
PCR	
aroE/M13F	TGTAAAACGACGGCCAGTcgtttagctgcagttgttgc
aroE/M13R	CAGGAAACAGCTATGACCcccacactggtggcattaac
ddl/M13F	TGTAAAACGACGGCCAGTttgccatggataaaacacgac
ddl/M13R	CAGGAAACAGCTATGACCcgcgcttgtcaaaactttcc
gdh/M13F	TGTAAAACGACGGCCAGTgtgctgaaaagattaaggtct
gdh/M13R	CAGGAAACAGCTATGACCtgcttccagctttatagtcatg
gki/M13F	TGTAAAACGACGGCCAGTggcattggaatgggatcacc
gki/M13R	CAGGAAACAGCTATGACCtctcccgcagctgacac
recP/M13F	TGTAAAACGACGGCCAGTgccaactcaggtcatccagg
recP/M13R	CAGGAAACAGCTATGACCttcgatagcagcatggatgg
spi/M13F	TGTAAAACGACGGCCAGTcgcttagaaaggtaagttatg
spi/M13R	CAGGAAACAGCTATGACCaggctgagattggtgattctc
xpt/M13F	TGTAAAACGACGGCCAGTggaggtcttatgaaattattag
xpt/M13R	CAGGAAACAGCTATGACCagatctgcctccttaaatac
Sequencing	
M13 F	TGTAAAACGACGGCCAGT
M13 R	CAGGAAACAGCTATGACC

### Genomic Data Analysis

We performed genomic data analysis for capsular sequence typing, PFGE, and MLST using Bionumerics version 7.6 software (Applied Maths). We retrieved MLST allelic profiles and STs from the *S. pneumoniae* MLST database (*29*) and submitted the new alleles and STs to the database curator for ST assignment. We compared MLST data with all Sp12F strains submitted to PubMLST.

### Statistical Analysis

We performed statistical analysis using SPSS version 14.0 software for Windows (IBM SPSS, Chicago, IL, USA). A p value <0.05 was considered statistically significant.

The data from the active surveillance during 2009–2016 are presented according to epidemiologic years, July through June. Each isolate was counted only once per episode; episodes were separated by >30 days for the same serotype or by any interval for different serotypes. Rate reductions and ratios were calculated. The following age groups were defined: <5 years, 5–17 years, 18–64 years, and ≥65 years of age.

## Results

**Figure 1:**
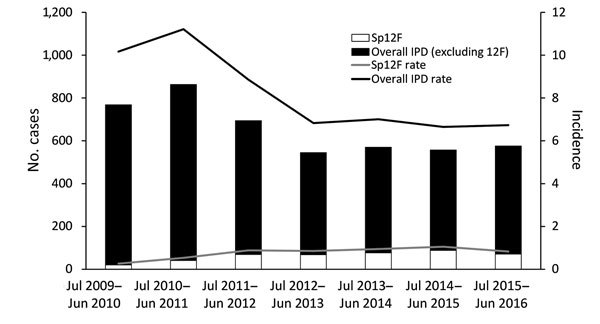
Overall invasive pneumococcal disease (IPD) and *Streptococcus pneumoniae* serotype 12F (Sp12F) cases and incidence (cases/100,000 population), Israel, July 2009–June 2016.

During the study, we identified 4,573 IPD episodes (93% bacteremia and 7% meningitis). Of those, 434 (9.5%) were caused by serotype 12F (Figure 1).

### Study Population Characteristics

Patients <5 years of age accounted for 27% of the overall study population; those 5–17 years of age, 8%; those 18–64 years of age, 31%; and those ≥65 years of age, 34%. Overall, 56.3% of all episodes occurred in male patients, and 86.4% of all episodes occurred among the Jewish population.

### Overall IPD

We ompared the first (2009–2010) and the last (2015–2016) study years and found that overall IPD rates (cases/100,000 population) declined by 34% (incidence rate ratio [IRR] 0.66, 95% CI 0.59–0.74), from 10.2 to 6.7 (Figure 1). IPD rates substantially declined in all age groups but most notably in children <5 years of age (48% rate reduction, from 30.9 to 16.1 cases/100,000 population). The results for the first study year (2009–2010) already showed a substantial reduction compared with the pre-PCV period (*15*,*16*) (Figure 1).

### IPD Caused by PCV13 Serotypes

Rates of IPD caused by PCV13 serotypes and proportions of all IPD episodes declined substantially throughout the study period. Rates (cases/100,000 population) of IPD caused by PCV7 serotypes declined by 90%, from 2.6 to 0.5, from the first to the last study years. Similarly, rates of IPD caused by PCV13 serotypes declined by 80%, from 7.4 to 1.5, during the study period.

Proportions of all IPD episodes caused by PCV7 serotypes substantially declined by 74%, from 24.8% in 2009–2010 to 6.4% in 2015–2016. Proportions of all IPD episodes caused by PCV13 serotypes decreased by 68%, from 69.4% to 22.4%.

### IPD Caused by Non-PCV13 Serotypes

IPD caused by non-PCV13 serotypes increased by 93%. Rates (cases/100,000 population) increased from 2.8 in 2009–2010 to 5.4 in 2015–2016.

### Sp12F

**Figure 2:**
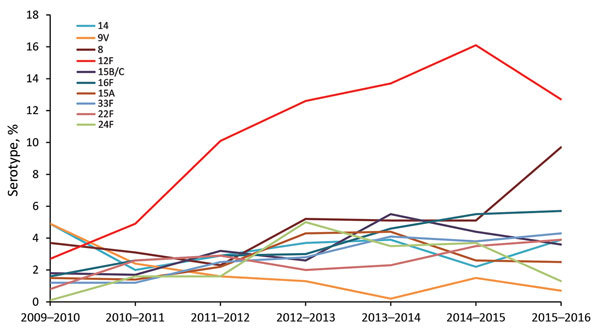
Predominant non–13-valent pneumococcal conjugate vaccine *Streptococcus pneumoniae* serotypes as proportions of overall invasive pneumococcal disease, Israel, July 2009–June 2016.

**Figure 3:**
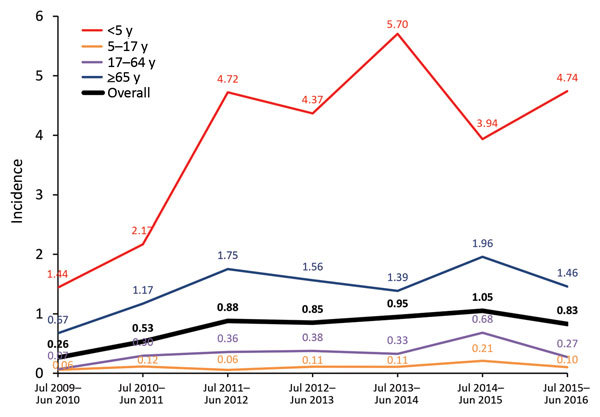
*Streptococcus pneumoniae* serotype 12F incidence (cases/100,000 population) by age group, Israel, July 2009–June 2016.

We observed a steady increase (except for the last study year) in the proportion of Sp12F out of all IPD episodes throughout the study: 2.7% in 2009–2010, 4.9% in 2010–2011, 10.1% in 2011–2012, 12.6% in 2012–2013, 13.7% in 2013–2014, 16.1% in 2014–2015, and 12.7% in 2015–2016 (Figure 2; Table 1). The incidence of IPD (in all ages) caused by Sp12F increased over the same period except for 1 slight decrease: 0.26 in 2009–2010, 0.53 in 2010–2011, 0.88 in 2011–2012, 0.85 in 2012–2013, 0.95 in 2013–2014, 1.05 in 2014–2015, and 0.83 in 2015–2016. Similar trends were observed in all age groups, with the sharpest increase observed in children <5 years of age, for which the respective figures by year were 1.44, 2.17, 4.72, 4.37, 5.70, 3.94, and 4.74 (Figure 3). Of note, 91.7% of all Sp12F episodes occurred in the Jewish population.

### Analysis of Sp12F Isolates

**Figure 4:**
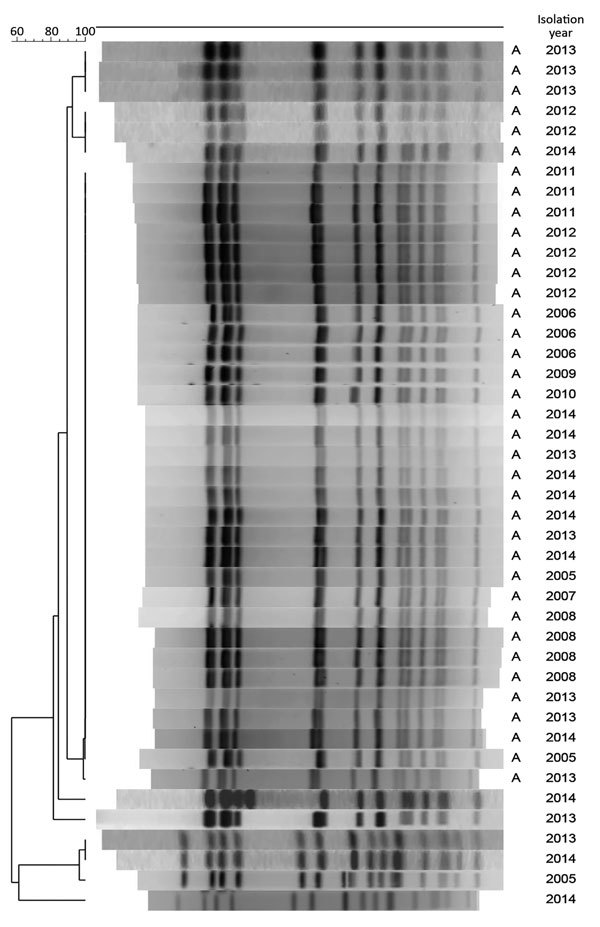
Pulsed-field gel electrophoresis analysis of a sample of *Streptococcus pneumoniae* serotype 12F isolates from Israel, 2000–2015. A indicates predominant pulsotypes.

**Figure 5:**
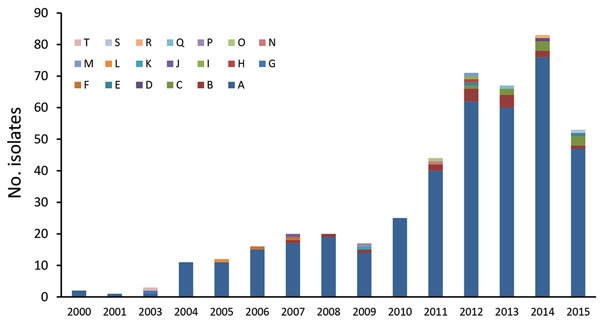
Pulsotype distribution of 445 *Streptococcus pneumoniae* serotype 12F isolates from Israel, by year, 2000–2015.

To assess the association between PCV7/PCV13 introduction and the dynamics of Sp12F rates, we analyzed strains isolated during 2000–2015. Overall, we analyzed 445 Sp12F strains by PFGE (Figure 4). The isolate population consists of a predominant pulsotype (A; 90.1% of isolates), and several other diverse pulsotypes (Figure 5). The predominant pulsotype was observed throughout the study period and was consistently prevalent among the Sp12F population; thus, the observed more general serotype Sp12F increase following PCV7/PCV13 introduction can be attributed to an expansion of this single clone during the study period.

### MLST of Sp12F

We further analyzed representative Sp12F isolates by MLST. Among 25 isolates with the predominant PFGE pulsotype, 24 were typed as ST3774 and 1 as the closely related ST3524. Four isolates from other PFGE pulsotypes were typed as ST989 and 1 isolate from another PFGE pulsotype was typed as ST3377.

**Figure 6:**
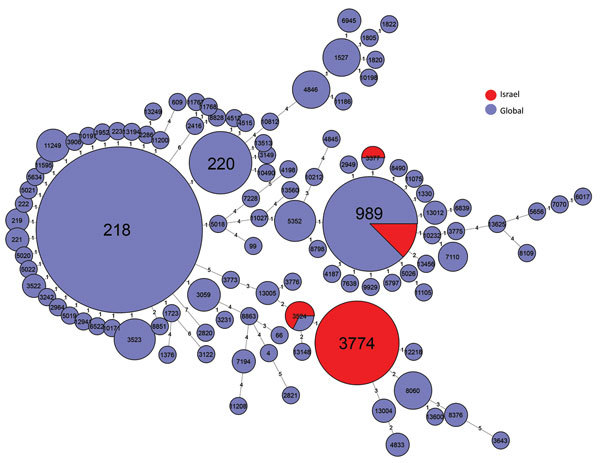
Multilocus sequence typing comparison of *Streptococcus pneumoniae* serotype 12F serotype isolates from Israel and globally.

We compared the MLST type with isolates reported as Sp12F in the global PubMLST database (*29*). This database contains 283 isolates serotyped as Sp12F and 105 sequence types. Among these isolates, the most common STs are ST218 and ST989, as previously published (*17*,*18*,*22*,*23*) (Figure 6). ST3774 is rare among isolates submitted to this global database, with only 1 isolate reported from Israel in 2006.

### Antimicrobial Drug Resistance of Sp12F

Among all Sp12F isolates, 89% were penicillin nonsusceptible (MIC >0.06); nonsusceptibility rates varied by year from 70% to 99% (MIC_50_ 0.12 μg/mL, range 0.02–1.2 μg/mL). However, most strains were fully susceptible to ceftriaxone (99%; MIC_50_ 0.06 μg/mL, range 0.02–1.0 μg/mL), clindamycin (95%), rifampin (100%), and vancomycin (100%).

## Discussion

*S. pneumoniae* serotype 12F, a non-PCV13 serotype, is currently the most prevalent pneumococcal serotype causing IPD in Israel. This previously rare serotype caused 434 IPD cases during July 2009–June 2016 and became prevalent in all age groups, most distinctly in young children. The population of Sp12F during 2000–2016 was >90% of the same clonal complex according to PFGE and MLST analyses, indicating the expansion of the ST3774 sequence type, which is currently unique to Israel. The predominant global Sp12F outbreak clones ST218 and ST989 (*21*) are very rare in IPD cases in Israel.

Sp12F was previously reported to have high invasive disease potential and is sometimes called hyperinvasive (*8*,*30*–*32*). It was reported as the most common serotype causing IPD in Japan (*31*,*32*), and it has been the cause of an IPD outbreak in Canada (*8*). Furthermore, several sites reported high disease potential for meningitis with Sp12F (*30*,*33*), and several Sp12F outbreaks have been reported in the literature among closed or crowded populations (*18*,*34*), including daycare centers (*35*), military groups (*34*), a jail (*20*), and a homeless shelter (*36*).

In a recent study from France that evaluated the invasive potential of specific serotypes and compared nasopharyngeal carriage in healthy children to carriage in children infected with IPD, Sp12F was shown to be a major cause of IPD but was not found in nasopharyngeal cultures (*30*). Furthermore, in Israel, Sp12F was rarely found in carriage studies before and after vaccination trials (*14*,*37*,*38*). These findings emphasize both the invasive nature of Sp12F and its failure to become a successful colonizer.

The emergence of IPD caused by Sp12F in Israel, mainly by clonal expansion, may be attributed, at least in part, to the observed more general serotype replacement after PCV implementation. The emergence of this serotype in the youngest age groups after PCV7/PCV13 introduction supports this hypothesis. However, the previously reported low carriage rate of Sp12F may counter this possibility, and current data on Sp12F carriage in Israel are needed to support this theory. Alternatively, a large-scale clonal outbreak, of long duration and high invasiveness rather than true replacement, may be at the origin of this serotype emergence.

Our results emphasize the role of Sp12F as a serious current public health challenge. Continued monitoring of IPD and specific serotype distribution are needed to enable the development of future vaccination strategies. The evolutionary origin of this emerging clone will be further studied by whole genome sequencing, which may offer insights into the virulence of Sp12F strains.
